# Skin carotenoid scores and metabolic syndrome in a general Japanese population: the Hisayama study

**DOI:** 10.1038/s41366-024-01575-7

**Published:** 2024-07-09

**Authors:** Yasumi Kimura, Jun Hata, Mao Shibata, Takanori Honda, Satoko Sakata, Yoshihiko Furuta, Emi Oishi, Takanari Kitazono, Toshiharu Ninomiya

**Affiliations:** 1 Department of Health and Nutrition, Faculty of Health Sciences, Hiroshima Shudo University, Hiroshima, Japan; 2 Department of Epidemiology and Public Health, Graduate School of Medical Sciences, Kyushu University, Fukuoka, Japan; 3 Department of Health Care Administration and Management, Kyushu University, Fukuoka, Japan; 4 Center for Cohort Studies, Graduate School of Medical Sciences, Kyushu University, Fukuoka, Japan; 5 Department of Medicine and Clinical Science, Graduate School of Medical Sciences, Kyushu University, Fukuoka, Japan

**Keywords:** Epidemiology, Lifestyle modification, Epidemiology, Nutrition

## Abstract

**Background:**

Higher vegetable intake is being promoted as an initiative to prevent lifestyle-related diseases. Carotenoids are yellow or red pigment components and are widely present in vegetables. Since ingested carotenoids accumulate in the skin, skin carotenoid levels are a quantitative indicator of vegetable intake. Recently, noninvasive optical sensors for assessing skin carotenoid levels were developed. We here examined the association between skin carotenoid scores measured using optical sensors and the presence of metabolic syndrome.

**Methods:**

A total of 1618 individuals (604 men and 1014 women) aged ≥ 40 years (mean age 63.1 years) participated in the study. Skin carotenoid scores were determined using a noninvasive optical sensor based on multiple spatially resolved reflectance spectroscopy. Metabolic syndrome was defined based on the Joint Scientific Statement criteria developed by six international scientific societies. Multivariable-adjusted logistic regression models were used.

**Results:**

The prevalence of metabolic syndrome was 31.3% (*n* = 506). A remarkably strong association was found between higher skin carotenoid scores and lower prevalence of metabolic syndrome after adjusting for confounders. The multivariable-adjusted odds ratio for the presence of metabolic syndrome in individuals with the highest quartile of skin carotenoid scores was 0.39 (95% confidence interval, 0.28–0.55) compared to those with the lowest quartile.

**Conclusions:**

Our findings suggest that higher skin carotenoid scores measured by non-invasive optimal sensors are significantly associated with a lower likelihood of having metabolic syndrome in the general Japanese population.

## Introduction

Metabolic syndrome (MetS) is characterized by the clustering of metabolic abnormalities that include central obesity and insulin resistance, dyslipidemia, elevated blood pressure, and impaired glucose tolerance [1]. Although not all research findings are consistent [2], observational studies have found that increasing vegetable intake is effective in preventing various lifestyle-related diseases such as obesity and MetS [3]. Therefore, the assessment of vegetable intake is important in promoting the prevention of obesity and MetS, but it is difficult to determine whether vegetable intake is adequate [4]. There are several dietary survey methods for assessing vegetable intake, such as diet records, diet recall, diet history, the duplicate method, and the food frequency method, but these methods tend to be technically complex and costly to accurately implement [5]. Carotenoids are yellow or red-pigmented compounds widely present in plants, particularly vegetables [6]. Carotenoid levels in serum are known to be useful biomarkers for quantitatively assessing individual levels of vegetable intake [7, 8]. However, such biomarkers require blood sampling for measurement and thus are not suitable for frequent assessment.

A noninvasive optical sensor based on the resonance Raman spectroscopy (RRS) method has been developed for assessing carotenoid levels in skin; this sensor has high precision but is relatively expensive [9–12]. Recently, two techniques based on the reflective spectrophotometry (RS) method have been developed to measure carotenoid levels through the skin [9–11, 13, 14]: the multiple spatially resolved reflection spectroscopy [9, 10, 14] and the pressure-mediated reflection spectroscopy [11, 13]. The carotenoid levels measured by these techniques have been shown to be highly correlated with those estimated by the RRS-based device, as well as highly correlated with serum carotenoid levels [9, 13, 15]. Several epidemiological studies conducted in Western and Asian countries have reported that participants with higher skin carotenoid levels estimated using an RRS-based device are at lower risk of having MetS [16, 17]. However, there is limited epidemiological evidence of an association between skin carotenoid scores estimated by RS-based devices and MetS. Herein, we examined the association between skin carotenoid scores determined using a noninvasive optical sensor with the multiple spatially resolved RS method and the presence of metabolic syndrome in a community-dwelling Japanese population.

## Methods

### Study population

**Fig. 1:**
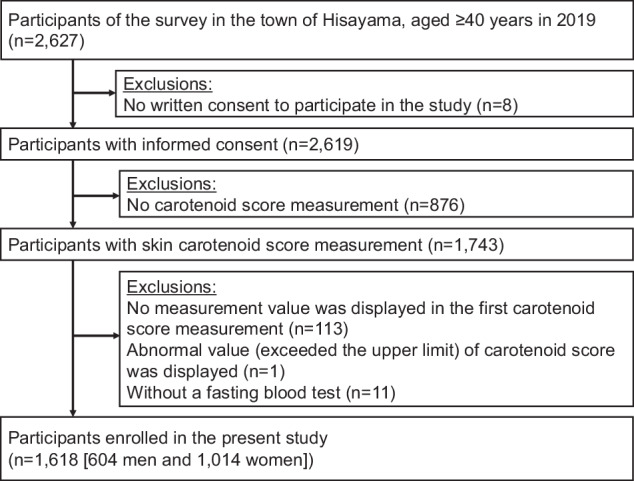
Flowchart of participant selection.

The present analysis was performed using the data from a cross-sectional survey of the Hisayama Study. The Hisayama Study is an ongoing population-based cohort study established in 1961 in the town of Hisayama, a suburb of the metropolitan area of Kyushu Island in Japan [18]. Since 1961, the age and occupational distributions and the nutrient intake of residents in this town have been similar to those of Japan as a whole based on data from the national census and nutrition survey [19]. Full community surveys of the health status of residents aged 40 and older have been repeated every one to two years since 1961 [20]. In 2019, a total of 2627 residents aged 40 and older underwent a screening examination. Of these, 2619 residents provided written consent to participate in the study. Skin carotenoid score measurement was included as an optional part of the screening examination, and 1743 participants (66.6%) who were interested in the carotenoid score measurement agreed to undergo this part of the screening. Then, after the exclusion of 114 participants in whom errors occurred in the carotenoid score measurement (113 participants for whom no measurement value was displayed in the first carotenoid score measurement and 1 for whom the skin carotenoid score exceeded the upper limit of 12.0) and 11 participants without a fasting blood test, the remaining 1618 participants (604 men and 1014 women) were enrolled in the present study (Fig. 1).

### Ethical approval

This study was conducted in accordance with the Declaration of Helsinki and the approval of the Kyushu University Institutional Review Board for Clinical Research (Approval No. 2023–56), and written informed consent was obtained from all the participants. All procedures were carried out in accordance with the relevant ethical guidelines.

### Measurement of skin carotenoid scores

To determine the skin carotenoid score, we used a multiple spatially resolved reflection spectroscopy sensor (Biozoom Services GmbH, Kassel, Germany) [9], because this sensor had been validated in a Japanese population at the time this study was conducted [15]. The sensor utilized 118 LED light emitters in 16 steps to provide light within the range of 350–1000 nm, while detecting the reflected light from 152 light-sensitive areas of the skin. To ensure the best possible correlation with the measured RRS values, an algorithm was developed to calculate the rank-order skin carotenoid score, which ranged from 0.1 to 12.0 [14]. Skin carotenoid scores determined by this system were reported to be significantly positively correlated with serum total carotenoid concentrations (*r* = 0.678) [15]. The skin carotenoid score was measured on the palm side of the thumb base, and the sensor was completely covered so that no stray light could enter. To ensure that the measurement conditions were the same for all participants, the values from the first measurement were used in this analysis.

### Diagnosis of metabolic syndrome

The definition of MetS was based on a Joint Scientific Statement issued by six international scientific societies [21]. Specifically, abdominal obesity was defined as a waist circumference ≥ 90 cm in men and ≥ 80 cm in women according to a WHO expert consultation for Asian populations [22]. Elevated blood pressure was defined as average systolic/diastolic blood pressure of ≥ 130/85 mmHg and/or current use of antihypertensive medications. Elevated blood glucose level was defined as fasting blood glucose of ≥ 5.6 mmol/L and/or current use of antidiabetic medication. Hypertriglyceridemia was defined as serum triglycerides of ≥ 1.69 mmol/L. Low high-density lipoprotein (HDL) cholesterolemia was defined as serum HDL cholesterol levels of < 1.03 mmol/L in men and of < 1.29 mmol/L in women. MetS was defined as the presence of 3 or more of these 5 components [21]. In sensitivity analyses, we defined MetS using other established criteria, namely the Japanese criteria [23] and the criteria of the International Diabetes Federation for Asians [24] (Table S1).

### Determination of other risk factors

All participants completed a self-administered questionnaire covering medical history, antidiabetic and antihypertensive medications, alcohol intake, smoking habits, and physical activity. Smoking habits and alcohol intake were categorized as current use or not. Regular exercise was defined as engaging in exercise ≥ 3 times per week during leisure time. Body height and weight were measured in light clothing without shoes and the body mass index (kg/m^2^) was calculated. The waist circumference was measured at the umbilical level in a standing position by a trained staff member. Blood pressure was measured three times using an automated sphygmomanometer with the participant seated after at least 5 min rest and the mean of the three measurements was calculated. Blood samples were collected from an antecubital vein after an overnight fast for the determination of blood glucose levels and lipids. Fasting blood glucose levels were measured by the hexokinase method. Serum concentrations of low-density lipoprotein (LDL) cholesterol, HDL cholesterol, and triglycerides were determined enzymatically.

### Statistical analysis

**Fig. 2:**
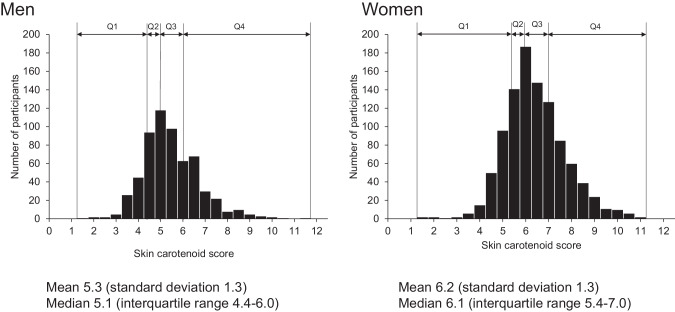
A histogram of the skin carotenoid scores. The skin carotenoid scores ranged from 1.4 to 11.2 (median, 5.1), with a mean of 5.3 and a standard deviation of 1.3 in men, and ranged from 1.4 to 10.7 (median, 6.1), with a mean of 6.2 and a standard deviation of 1.3 in women.

Skin carotenoid scores were divided into four categories based on the sex-specific quartile distribution because skin carotenoid scores were higher in women than in men (Fig. 2). The trends in the mean values or the frequencies of cardiovascular risk factors across the sex-specific quartiles of skin carotenoid scores were tested by linear regression analysis or logistic regression analysis, respectively. For serum triglycerides, the median values and their interquartile ranges for each skin carotenoid score are shown due to the skewed distributions, and their trends across skin carotenoid scores were tested by a Jonckheere–Terpstra trend test. Logistic regression analysis was used to estimate adjusted odds ratios (ORs) with 95% confidence intervals (CIs) of MetS according to the skin carotenoid scores. The linear trends in the risk estimates were tested using a logistic regression model including the skin carotenoid quartiles represented as ordinal numbers (0, 1, 2, and 3) and the relevant covariates. We also used restricted cubic splines to show the shape of these associations with 5 knots placed at the 5th, 25th, 50th, 75th and 95th percentiles of skin carotenoid scores (3.8, 4.9, 5.8, 6.7 and 8.5, respectively) [25]. The 5th percentile was chosen as the reference value. To examine whether there was a need for cubic spline terms in addition to a linear term, the non-linearity for the association was tested by using a likelihood ratio test comparing the relevant model with only a linear term against the model with linear and cubic spline terms [25]. In the subgroup analysis of potential confounding factors, participants were divided into two groups by the sex-specific median values of skin carotenoid score, and the multivariable-adjusted ORs for the presence of MetS in the group with higher skin carotenoid score against the groups with lower scores were calculated for each subgroup. The heterogeneity in the associations across subgroups was tested by adding the multiplicative interaction term in the relevant logistic model. All statistical analyses were performed using SAS 9.4 software (SAS Institute, Cary, NC). In all analyses, two-sided *p*-values (*P* < 0.05) were considered to indicate statistical significance.

## Results

A histogram of the skin carotenoid scores is shown in Fig. 2. In men, the skin carotenoid scores ranged from 1.4 to 11.2 (median, 5.1), with a mean of 5.3 and standard deviation of 1.3. In women, it ranged from 1.4 to 10.7 (median, 6.1), with a mean of 6.2 and a standard deviation of 1.3. The distribution of the carotenoid score was approximately normal, with skewness of 0.436, 0.785, and 0.365 and kurtosis of 0.610, 1.578, and 0.779 for the total study population (*n* = 1618), men (*n* = 604), and women (*n* = 1014), respectively. The clinical characteristics of participants according to the sex-specific quartiles of skin carotenoid scores are summarized in Table 1. Participants with higher skin carotenoid scores were more likely to be older. Higher skin carotenoid scores were associated with lower mean values of body mass index, waist circumference, diastolic blood pressure, and serum triglycerides, and the proportions of participants with current smoking and current alcohol drinking decreased with increasing skin carotenoid scores, whereas the mean values of serum HDL cholesterol and the proportions of participants with use of lipid-modifying agents and regular exercise increased with increasing skin carotenoid scores. Among the MetS components, the proportions of abdominal obesity and hypertriglyceridemia decreased significantly with higher skin carotenoid scores.

**Table 1:** Clinical characteristics of the study participants according to sex-specific quartiles of skin carotenoid scores.

Characteristic	Quartiles of skin carotenoid scores				
	Q1	Q2	Q3	Q4	*P-* trend
	M: ≤ 4.4 W: ≤ 5.3 (*n* = 407)	M: 4.5–5.0 W: 5.4–6.0 (*n* = 388)	M: 5.1–6.0 W: 6.1–6.9 (*n* = 414)	M: ≥ 6.1 W: ≥ 7.0 (*n* = 409)	
Age (years)^a^	59.1 (12.4)	62.5 (11.9)	63.3 (12.5)	67.4 (11.0)	<0.001
Women (%)	62.2	64.2	61.1	63.3	0.96
Body mass index (kg/m^2^)	23.9 (3.9)	23.4 (3.6)	23.0 (3.3)	22.4 (3.2)	<0.001
Waist circumference (cm)	86.3 (10.9)	85.4 (9.7)	84.1 (9.0)	83.0 (8.9)	<0.001
Systolic blood pressure (mmHg)	124.9 (18.2)	124.8 (16.5)	123.4 (18.7)	124.0 (19.6)	0.30
Diastolic blood pressure (mmHg)	72.9 (12.4)	72.4 (11.2)	69.7 (11.8)	69.7 (12.0)	<0.001
Use of antihypertensive agent (%)	33.4	37.1	35.8	36.2	0.51
Fasting plasma glucose (mmol/L)	5.81 (1.01)	5.80 (0.99)	5.78 (0.88)	5.71 (0.86)	0.14
Use of glucose-lowering agents (%)	8.9	8.8	7.3	9.3	0.98
Serum LDL cholesterol (mmol/L)	3.10 (0.84)	3.20 (0.85)	3.16 (0.85)	3.09 (0.80)	0.71
Serum HDL cholesterol (mmol/L)	1.72 (0.46)	1.71 (0.46)	1.77 (0.44)	1.81 (0.48)	0.001
Serum triglycerides (mmol/L)^b^	1.11 (0.79–1.56)	1.16 (0.81–1.59)	1.01 (0.75–1.38)	1.03 (0.77–1.38)	0.001
Use of lipid-modifying agents (%)	21.2	23.7	23.7	32.3	<0.001
Current smoking (%)	21.1	11.6	7.7	4.9	<0.001
Current alcohol drinking (%)	55.8	51.0	51.5	46.2	0.01
Regular exercise (%)	11.6	16.8	19.6	26.7	<0.001

*M* men, *W* women, *Q* quartile, *LDL* low-density lipoprotein, *HDL* high-density lipoprotein.

^a^Values are expressed as the mean (standard deviation) or a percentage.

^b^Median values and interquartile ranges of serum triglycerides are shown due to the skewed distributions, and the trend was tested by a Jonckheere-Terpstra trend test.

The overall prevalence of MetS (defined by the Joint Scientific Statement criteria) was 31.3% in the analyzed participants. As shown in Table 2, the age- and sex-adjusted OR for the presence of the MetS decreased significantly with increasing skin carotenoid scores (*P*-trend < 0.001). This significant downward association remained significant after adjusting for age, sex, serum LDL cholesterol, use of lipid-modifying agents, current smoking, current alcohol drinking, and regular exercise (*P*-trend < 0.001). The multivariable-adjusted OR for the presence of MetS was significantly lower in participants in the highest quartile of skin carotenoid scores than in those in the lowest quartile (OR 0.39; 95% CI, 0.28–0.55). With regard to MetS components, higher skin carotenoid scores were significantly associated with lower multivariable-adjusted ORs for the presence of abdominal obesity (*P*-trend < 0.001), elevated blood pressure (*P*-trend < 0.001), elevated blood glucose (*P*-trend < 0.001) low HDL cholesterolemia (*P*-trend = 0.048), and hypertriglyceridemia (*P*-trend = 0.004). When BMI was considered as an adjustment factor for MetS components other than abdominal obesity, the association of skin carotenoid levels with the presence of elevated blood pressure and elevated blood glucose was still significant, but the magnitude of the association was attenuated. In addition, the association of skin carotenoid levels with the presence of low HDL cholesterolemia and hypertriglyceridemia did not reach the level of statistical significance (Table 2). As a sensitivity analysis, we examined the association between skin carotenoid scores and metabolic syndrome using other criteria for MetS (i.e., the Japanese criteria and the International Diabetes Federation criteria). Both these criteria yielded inverse associations similar to those obtained using the Joint Scientific Statement criteria (Table S2).

**Table 2:** Adjusted odds ratios for metabolic syndrome and its components according to the sex-specific quartiles of the skin carotenoid scores.

	Quartiles of skin carotenoid scores				*P-* trend
	Q1	Q2	Q3	Q4	
	M: ≤ 4.4 W: ≤ 5.3 (*n* = 407)	M: 4.5–5.0 W: 5.4–6.0 (*n* = 388)	M: 5.1–6.0 W: 6.1–6.9 (*n* = 414)	M: ≥ 6.1 W: ≥ 7.0 (*n* = 409)	
Metabolic syndrome^a^					
Number of participants with MetS	140 (34.4%)	141 (36.3%)	125 (30.2%)	100 (24.4%)	
Age- and sex-adjusted OR (95% CI)	1.00 (reference)	0.94 (0.70–1.27)	0.68 (0.50–0.92)	0.42 (0.30–0.58)	<0.001
Multivariable-adjusted OR (95% CI)^b^	1.00 (reference)	0.91 (0.66–1.24)	0.64 (0.47–0.89)	0.39 (0.28–0.55)	<0.001
Metabolic syndrome components					
Abdominal obesity					
Number of participants with abdominal obesity	226 (55.5%)	219 (56.4%)	220 (53.1%)	185 (45.2%)	
Age- and sex-adjusted OR (95% CI)	1.00 (reference)	0.91 (0.68–1.23)	0. 80 (0.60–1.08)	0.48 (0.35–0.65)	<0.001
Multivariable-adjusted OR (95% CI)^b^	1.00 (reference)	0.84 (0.62–1.14)	0.75 (0.55–1.02)	0.45 (0.33–0.62)	<0.001
Elevated blood pressure					
Number of participants with elevated blood pressure	217 (53.3%)	227 (58.5%)	231 (55.8%)	226 (55.3%)	
Age- and sex-adjusted OR (95% CI)	1.00 (reference)	0.92 (0.66–1.27)	0.71 (0.51–0.98)	0.45 (0.33–0.63)	<0.001
Multivariable-adjusted OR (95% CI)^b^	1.00 (reference)	0.93 (0.66–1.30)	0.73 (0.52–1.03)	0.43 (0.30–0.61)	<0.001
Multivariable-adjusted OR (95% CI)^c^	1.00 (reference)	1.01 (0.71–1.42)	0.87 (0.62–1.23)	0.54 (0.38–0.78)	<0.001
Elevated blood glucose					
Number of participants with elevated blood glucose	217 (55.3%)	218 (56.2%)	216 (52.2%)	203 (49.6%)	
Age- and sex-adjusted OR (95% CI)	1.00 (reference)	0.99 (0.74–1.34)	0.78 (0.58–1.04)	0.59 (0.44–0.80)	<0.001
Multivariable-adjusted OR (95% CI)^b^	1.00 (reference)	0.98 (0.72–1.33)	0.75 (0.56–1.02)	0.56 (0.41–0.77)	<0.001
Multivariable-adjusted OR (95% CI)^c^	1.00 (reference	1.08 (0.79–1.48)	0.90 (0.66–1.24)	0.74 (0.53–1.02)	0.04
Low HDL cholesterolemia					
Number of events participants with low HDL cholesterolemia	38 (9.3%)	37 (9.5%)	31 (7.5%)	25 (6.1%)	
Age- and sex-adjusted OR (95% CI)	1.00 (reference)	1.00 (0.62–1.62)	0.78 (0.47–1.28)	0.61 (0.35–1.04)	0.046
Multivariable-adjusted OR (95% CI)^b^	1.00 (reference)	0.93 (0.57–1.54)	0.75 (0.44–1.26)	0.60 (0.34–1.04)	0.048
Multivariable-adjusted OR (95% CI)^c^	1.00 (reference	1.11 (0.66–1.85)	1.01 (0.59–1.73)	0.90 (0.50–1.62)	0.69
Hypertriglyceridemia					
Number of participants with hypertriglyceridemia	88 (21.6%)	76 (19.6%)	60 (14.5%)	59 (14.4%)	
Age- and sex-adjusted OR (95% CI)	1.00 (reference)	0.88 (0.62–1.25)	0.60 (0.42–0.86)	0.59 (0.41–0.87)	0.001
Multivariable-adjusted OR (95% CI)^b^	1.00 (reference)	0.88 (0.61–1.26)	0.62 (0.42–0.90)	0.62 (0.42–0.92)	0.004
Multivariable-adjusted OR (95% CI)^c^	1.00 (reference	0.97 (0.67–1.42)	0.73 (0.50–1.09)	0.84 (0.56–1.27)	0.21

*M* men, *W* women, *Q* quartile, *OR* odds ratio, *CI* confidence interval, *LDL* low-density lipoprotein, *HDL* high-density lipoprotein.

^a^The definition of MetS was based on the Joint Scientific Statement by six international scientific societies.

^b^Adjusted for age, sex, serum LDL cholesterol, lipid-modifying agents, current smoking, current alcohol drinking and regular exercise.

^c^Adjusted for age, sex, serum LDL cholesterol, lipid-modifying agents, current smoking, current alcohol drinking, regular exercise and body mass index.

**Fig. 3:**
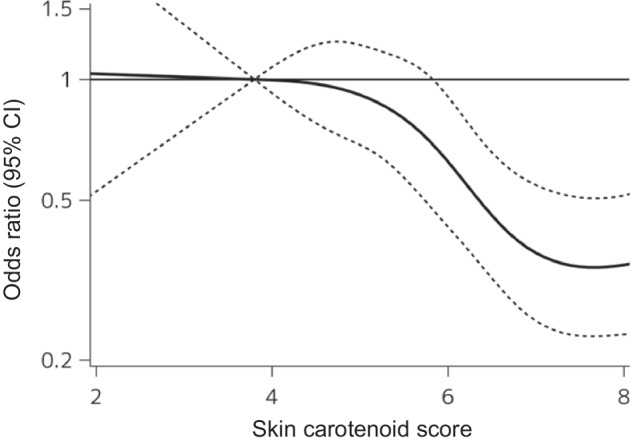
Restricted cubic splines for the association of skin carotenoid scores with the multivariable-adjusted odds ratios for the presence of metabolic syndrome. Solid lines represent the hazard ratio, and dashed lines represent the 95% Cl of the odds ratio. Knots were placed at the 5th, 25th, 50th, 75th, and 95th percentiles (3.8, 4.9, 5.8, 6.7, and 8.5) of skin carotenoid levels. A reference point was set at the 5th percentile (3.8 for skin carotenoid levels). The *X*-axis in the graph shows up to the 90th percentile value of skin carotenoid levels (8.0). The risk estimates were adjusted for age, sex, serum total cholesterol, use of lipid-modifying agents, current alcohol drinking, current smoking, and regular exercise.

As shown in Fig. 3, we observed a non-linear association of skin carotenoid scores with MetS by using a restricted cubic spline model (*P* non-linearity = 0.004). The multivariable-adjusted ORs for the presence of MetS tended to decrease at around skin carotenoid scores of 5. Compared to the reference value (3.8 for skin carotenoid score), the upper limits of the 95% confidence interval were below 1.0 at a carotenoid score of around 6. Finally, we compared the multivariable-adjusted ORs for the presence of MetS in the groups below the median of carotenoid score against the group above the median score across the subgroups of potential cofounding factors (Table 3). We found no evidence of significant heterogeneity in the associations between the subgroups of these factors (all *P*-heterogeneity > 0.26).

**Table 3:** Subgroup analysis by potential confounding factors for the association between skin carotenoid score and the presence of metabolic syndrome.

Variables	Number of events/subjects	OR (95% CI) for higher score group vs. lower score group ^a,b^	*P* value	*P* for heterogeneity
Overall	496/1603	0.54 (0.43–0.68)	<0.001	
Age				
< 65 years	176/770	0.45 (0.31–0.66)	<0.001	0.62
≥ 65 years	320/833	0.60 (0.44–0.80)	<0.001	
Sex				
Men	174/593	0.66 (0.45–0.96)	0.03	0.41
Women	322/1010	0.47 (0.34–0.63)	<0.001	
Serum LDL cholesterol				
< 3.62 mmol/L	339/1179	0.53 (0.40–0.70)	<0.001	0.26
≥ 3.62 mmol/L	157/424	0.52 (0.34–0.81)	0.004	
Current Smoking				
No	449/1425	0.52 (0.41–0.66)	<0.001	0.46
Yes	47/178	0.74 (0.33–1.66)	0.47	
Current alcohol drinking				
No	261/790	0.51 (0.37–0.71)	<0.001	0.39
Yes	235/813	0.56 (0.40–0.79)	<0.001	
Regular exercise				
< 3 times/week	401/1305	0.53 (0.41–0.69)	<0.001	0.91
≥ 3 times/week	95/298	0.58 (0.34–1.00)	0.05	

*OR* odds ratio, *CI* confidence interval, *LDL* low-density lipoprotein.

^a^Skin carotenoid scores were divided into two categories based on the sex-specific median.

^b^Adjusted for age, sex, serum LDL cholesterol, lipid-modifying agents, current smoking, current alcohol drinking, and regular exercise.

## Discussion

The present cross-sectional study demonstrated that higher skin carotenoid levels as estimated by using a noninvasive optical sensor and multiple spatially resolved RS were significantly associated with a lower risk of the presence of MetS and its components in a community-dwelling Japanese population. Previous epidemiological studies have also reported a significant inverse association between skin carotenoid levels and the presence of MetS [16, 17, 26]. A hospital-based study in the United States [16] and a population-based study conducted in Singapore [17] showed that a lower skin carotenoid level estimated by the RRS method was associated with a higher prevalence of MetS. Another cross-sectional study of Japanese adults using health check-up data reported that skin carotenoid levels estimated by the RS method using the same optimal sensor as in the present study were positively correlated with vegetable intakes and negatively correlated with MetS components and cardiovascular risk factors [15], and the multivariable-adjusted OR for the presence of MetS decreased with higher skin carotenoid levels [26]. Both these previous findings and our present study indicated an association between skin carotenoid levels and a lower risk of the presence of MetS. Intriguingly, the present study showed that the OR for the presence of MetS started to decrease from a skin carotenoid score of around 5, which may suggest that a certain level of carotenoid intake is required in order to realize the risk reduction for developing MetS. Although to our knowledge there are no comparable previous studies on the association between MetS and skin carotenoid scores using this cut point, we believe that skin carotenoid levels estimated by the noninvasive optical sensor may be useful in assessing the daily vegetable intakes and in providing dietary guidance for individuals for the prevention of MetS.

The possible mechanisms underlying the significant inverse association between the skin carotenoid level and the presence of MetS in the present study should be discussed. Skin carotenoid levels have been reported to reflected serum total carotenoid levels [15]. Carotenoids are potent natural antioxidants [27]. Oxidative stress can be accumulated in the form of damage to proteins, lipids, and carbohydrates and is considered to disrupt redox signaling pathways in cells, leading to insulin resistance and consequently MetS [28]. Carotenoids can act as direct antioxidants, quenching singlet oxygen and reducing the formation of lipid peroxides [27, 29]. A study of 22 overweight Korean women demonstrated that a high-vegetable and fruit diet led to decreases in markers of oxidative stress, including interleukin-6, and increases in serum carotenoid levels [30]. Therefore, the antioxidant effect of carotenoids in the human body may contribute to a reduction in the risk of developing MetS. On the other hand, the present study found an association between skin carotenoid levels and the odds ratios for MetS components after adjustment for BMI. Skin carotenoid levels may reflect healthy dietary behaviors (e.g., diverse dietary patterns, regular eating habits, and slow eating speed), which are thought to prevent obesity and subsequent MetS, rather than a physiological response to carotenoids themselves [31–33].

Several limitations in the present study should be noted. First, because this is a cross-sectional study, the findings of the present study do not support the causality of the observed associations. Second, there is a possibility of selection bias due to non-participation. Approximately 30% of study individuals were excluded from the analysis because they did not wish to participate in the skin carotenoid score measurements. Third, participants in this study might have voluntarily measured their skin carotenoid scores prior to the study and received feedback on their scores. However, at the time this study was conducted, the instrument could not be used for anything other than research purposes, so this possibility is unlikely. Finally, it was difficult to fully address the influence of dietary factors (e.g., total energy intake and dietary intakes of vegetable and nutrients) on the association between skin carotenoid levels and prevalence of MetS because we did not conduct a dietary survey in the 2019 health examination. In addition, the possibility of residual confounding factors such as dietary behaviors and health literacy could not be ruled out, although there was no significant heterogeneity in the associations between subgroups of lifestyle factors, including current smoking, current alcohol drinking, and regular exercise.

## Conclusions

Our findings suggest that higher skin carotenoid scores measured by non-invasive optimal sensors are significantly associated with a lower likelihood of having metabolic syndrome in the general Japanese population.

## Supplementary information

Supplementary Tables

## Data Availability

The data described in the manuscript, the code book, and the analytic code will not be made available because they contain confidential clinical and demographic data of the study participants. However, further information about the datasets is available with the permission of the principal investigator of the Hisayama Study (TN) on reasonable request for purposes of replicating procedures and results.
