# Supplementary material for: Skin carotenoid scores and metabolic syndrome in a general Japanese population: the Hisayama study
Source: Int J Obes (Lond). 2024 Jul 9;48(10):1465–71. doi: 10.1038/s41366-024-01575-7 (PMC11420057; doi:10.1038/s41366-024-01575-7)
Supplement: Supplementary file 1 — Supplementary Tables [file 41366_2024_1575_MOESM1_ESM.pdf]

Supplementary Table 1. Metabolic syndrome criteria used in the present study

	Joint Scientific Statement from IDF/NHLBI/AHA/WHF/IAS/ IASO with the Asian cutoff for waist circumference [a]	Japanese criteria [b]	IDF criteria for Asians [c]
Definition of metabolic syndrome	Three or more of components (1)–(5) listed below	Component (1) plus any two or more of components (2)–(4) listed below	Component (1) plus any two or more of components (2)–(5) listed below
Metabolic syndrome components			
Abdominal obesity	(1) Waist circumference ≥90 cm (men), ≥80 cm (women)	(1) Waist circumference ≥85 cm (men), ≥90 cm (women)	(1) Waist circumference ≥90 cm (men), ≥80 cm (women)
Elevated blood pressure	(2) Blood pressure ≥130/85 mmHg and/or antihypertensive agents	(2) Blood pressure ≥130/85 mmHg and/or antihypertensive agents	(2) Blood pressure ≥130/85 mmHg and/or antihypertensive agents
Elevated fasting plasma glucose	(3) Fasting plasma glucose ≥5.6 mmol/L and/or glucose-lowering agents	(3) Fasting plasma glucose ≥6.1 mmol/L and/or glucose-lowering agents	(3) Fasting plasma glucose ≥5.6 mmol/L and/or glucose-lowering agents
Dyslipidemia	(4) Serum HDL cholesterol <1.03 mmol/L (men), <1.29 mmol/L (women)<br>(5) Serum triglycerides ≥1.69 mmol/L	(4) Serum HDL cholesterol <1.03 mmol/L, and/or serum triglycerides ≥1.69 mmol/L	(4) Serum HDL cholesterol <1.03 mmol/L (men), <1.29 mmol/L (women)<br>(5) Serum triglycerides ≥1.69 mmol/L

Abbreviations: IDF, International Diabetes Federation; NHLBI, National Heart, Lung, and Blood Institute; AHA, American Heart Association; WHF, World Heart Federation; IAS, International Atherosclerosis Society; IASO, International Association for the Study of Obesity; HDL, high density lipoprotein.

[a] Refer to reference 21.

[b] Refer to reference 23.

[c] Refer to reference 24.

Supplementary Table 2. Multivariable-adjusted odds ratios for metabolic syndrome according to the quartile of skin carotenoid scores

	Japanese criteria [a]					IDF criteria for Asians [b]				
	Skin carotenoid score levels					Skin carotenoid score levels				
	Q1	Q2	Q3	Q4		Q1	Q2	Q3	Q4	
	M: ≤4.4<br>W: ≤5.3<br>(n = 407)	M: 4.5–5.0<br>W: 5.4–6.0<br>(n = 388)	M: 5.1–6.0<br>W: 6.1–6.9<br>(n = 414)	M: ≥6.1<br>W: ≥7.0<br>(n = 409)	*p*- trend	M: ≤4.4<br>W: ≤5.3<br>(n = 407)	M: 4.5–5.0<br>W: 5.4–6.0<br>(n = 388)	M: 5.1–6.0<br>W: 6.1–6.9<br>(n = 414)	M: ≥6.1<br>W: ≥7.0<br>(n = 409)	*p*- trend
**Metabolic syndrome**										
Number of participants with MetS	78 (19.2%)	80 (20.6%)	62 (15.0%)	45 (11.0%)		132 (32.4%)	130 (33.5%)	110 (26.6%)	89 (21.8%)	
Age- and sex-adjusted OR (95% CI)	1.00 (reference)	1.01 (0.70–1.47)	0.60 (0.41–0.89)	0.37 (0.24–0.57)	<0.001	1.00 (reference)	0.89 (0.66–1.22)	0.61 (0.44–0.83)	0.38 (0.27–0.54)	<0.001
Multivariable-adjusted OR (95% CI) [c]	1.00 (reference)	0.99 (0.68–1.44)	0.58 (0.39–0.86)	0.34 (0.22–0.53)	<0.001	1.00 (reference)	0.84 (0.61–1.15)	0.57 (0.41–0.80)	0.36 (0.25–0.50)	<0.001

Abbreviations: M, men; W, women; Q, quartile; OR, Odds ratio; CI, Confidence interval, IDF, International Diabetes Federation.

[a] Refer to reference 23.

[b] Refer to reference 24.

[c] Adjusted for age, sex, serum LDL cholesterol, lipid-modifying agents, current smoking, current alcohol drinking, and regular exercise.
